# Gaps in the Engagement of People With Lived and Living Experience and Caregivers in Mental Health and Substance Use Health Research: A Qualitative Study of Untapped Potential

**DOI:** 10.1111/hex.70393

**Published:** 2025-08-14

**Authors:** Lisa D. Hawke, Jingyi Hou, Charlotte Munro, Claudia Sendanyoye, Shoshana Hauer, Mary Rose van Kesteren, Katie Upham, Tanya Halsall, Yona Lunsky

**Affiliations:** ^1^ Centre for Addiction and Mental Health Toronto Ontario Canada; ^2^ University of Toronto Toronto Ontario Canada; ^3^ University of Ottawa Institute of Mental Health Research Ottawa Ontario Canada

**Keywords:** engagement, lived/living experience, mental health, patient‐oriented research, substance use health

## Abstract

**Background:**

People with lived/living experience and family/caregivers (PLLEX‐C) can be engaged in mental health and substance use health research in roles such as advisors, collaborators, and co‐researchers. While there is a substantial body of research describing the barriers and facilitators to effective lived experience engagement, the actual contributions that PLLEX‐C are making to the research remains under‐explored. This qualitative descriptive study aimed to explore new areas where PLLEX‐C can contribute to the research process. We wanted to understand gaps in the contributions of PLLEX‐C and how we can provide opportunities to grow and enhance their contributions to the research in which they are engaged.

**Methods:**

A Canada‐wide sample of 28 PLLEX‐C took part in one of five focus groups, while 12 researchers from across Canada took part in individual interviews using a co‐designed semi‐structured interview guide. Discussions were recorded, transcribed, and analyzed using codebook thematic analysis. We engaged PLLEX‐C throughout the course of the study.

**Results:**

Gaps in the engagement of PLLEX‐C were found across the research lifecycle. This included key aspects of project initiation, like establishing research questions and priorities, contributing to grant applications, and contributing to ethics processes. Gaps were also encountered in the research operations process, in terms of recruitment processes and data analysis. Lastly, gaps at the end‐of‐grant knowledge translation stage included manuscript co‐authorship and co‐presentation at conferences or other events.

**Conclusions:**

PLLEX‐C are willing to be engaged in research across the research lifecycle, but many have experienced areas of untapped potential. To develop appropriate engagement plans for a given project, it is important to have open discussions with the PLLEX‐C engaged to understand their areas of skill, interest, and professional development goals, as well as barriers to full engagement in some stages of the project. This will make it possible to co‐design a creative and flexible personalized engagement plan that is meaningful to them and maximizes their engagement potential. This process will ensure that authentic engagement overrides tokenistic practices.

**Patient Engagement:**

This study was conducted on lived/living experience engagement and the team includes people with lived/living experience. A lived/living experience working group contributed to the design and operation of the project, as well as to data analysis, interpretation, and co‐authorship.

## 1 Background

Historically, research paradigms have considered people with health conditions and their family members or caregivers to be subjects worthy of study. Under today's growing research paradigm, they are colleagues who can contribute to the research and enhance it by sharing their perspectives derived from lived/living experience with the health condition and their use of the healthcare system [1]. From an experiential perspective, people living with health conditions can provide substantial, meaningful contributions to research activities about their conditions and should be engaged in these ways. Indeed, their engagement in research democratizes the research process and increases its relevance by taking it out of the lab and into the real world [2], for a process that benefits the researchers, the people engaged, and society as a whole.

Lived experience engagement is increasingly common across the health sciences, and has been particularly called for in mental health and substance use health research [3]. While often referred to as ‘patient engagement’ or ‘patient and public involvement’, in mental health and substance use health research, our colleagues have preferred to be referred to as people with lived and living experience (PLLEX) and family members or caregivers (‐C). Lived experience engagement may be particularly important in mental health and substance use health research for a number of reasons. These include the past and present inequities in care; the unbalanced power dynamics associated with involuntary or paternalistic care cultures; the pervasive stigma about mental health and substance use health that exists in society, in families, in healthcare settings, and in research; and the important impact of social and structural determinants of health on mental health and substance use health [3, 4, 5]. Note that mental health and substance use‐related experiences frequently co‐occur and are increasingly addressed together [6]; PLLEX‐C engagement, therefore, often includes people who can speak to both categories of health together.

PLLEX‐C can contribute to the full spectrum of research studies, from preclinical research to knowledge mobilization, including integrated knowledge translation approaches in which they are engaged throughout the research lifecycle [7, 8, 9, 10, 11]. PLLEX‐C can make a wide range of contributions to research projects [12], such as defining research priorities and establishing research questions at the early stage of the research; co‐designing procedures and study pathways, developing and refining participant‐facing materials, contributing to recruitment plans, collecting data, analyzing data, and interpreting findings during study operations; and co‐producing and co‐presenting both lay and academic knowledge translation materials at the end‐of‐grant period. Specific contributions may depend on the model of the engagement, from informing to empowering [13]. Contributions may also depend to a degree on the type of research conducted, including preclinical research [7], knowledge synthesis studies [14], clinical trials [8], quantitative research [15], qualitative research [16] and other forms of research.

A review paper shows that PLLEX‐C contributions have positive impacts on the research, providing greater reflexivity, improved research methods, better recruitment, and more depth to the data [3]. Impacts on the research environment include greater diversity, balancing of power dynamics, and destigmatization of research, among others. There are further positive impacts observed on the researchers (e.g., new perspective, strengthened commitment), and to the PLLEX‐C involved (e.g., confidence, knowledge, skills, agency). However, engagement must be comprehensive and authentic to achieve the full spectrum of positive impacts across the phases of the research. In addition, the evaluation of the concrete impacts of engagement remains a key evidence gap in the emerging science of lived experience engagement, with evaluations derived largely from commentaries and personal perspectives rather than rigorous research processes [17]. The literature highlights that a variety of forms of evaluation of engagement are urgently needed, and these evaluations should be co‐designed with PLLEX‐C [17]. Multiple methodologies should be used to get a clear picture of various aspects of the engagement process, such as the way lived experience engagement impacts the research conducted and those involved in the engagement process [17].

There is a substantial body of research describing the barriers and facilitators to authentic engagement in mental health and substance use health research [3]. Barriers are seen across the researchers, the team, the institution, and the PLLEX‐C themselves, but the barriers are reflected by corresponding facilitators that can be implemented to help teams conduct engagement effectively. However, inconsistent reporting leaves an evidence gap around the actual impact that PLLEX‐C make to research [3]. Most manuscripts do not follow reporting guidelines to describe the engagement experience, which leaves reporting limited and subjective [3, 18]. In our experience, PLLEX‐C frequently express that they want to and are able to contribute more than they typically do across multiple research projects and as members of various research teams. This is reflected in the literature, which suggests the level of engagement to date is often limited, tokenistic, and insufficient [3, 12, 19]. Further research is required to identify where the gaps are in lived experience engagement and where PLLEX‐C can contribute more fulsomely to the research process.

This qualitative descriptive study responds to this evidence and practice gap. We aimed to understand what new areas PLLEX‐C can contribute to in research projects, from the perspectives of both PLLEX‐C and researchers. This included understanding gaps in the contributions that PLLEX‐C typically have the opportunity to make to research, which will help us understand how we might be able to provide them with opportunities to grow and expand their contributions to the research in which they are engaged.

## 2 Methods

This qualitative study was conducted according to a qualitative descriptive and pragmatic approach, with lived experience engagement embedded in all stages [20]. The Standards for Reporting Qualitative Research (SRQR) reporting guidelines (Appendix A) are used to report on the study [21].

### 2.1 Participants and Recruitment

A sample of 28 PLLEX‐C from across Canada took part in one of five focus groups, while a sample of 12 researchers from across Canada took part in individual interviews. To be eligible for the study, participants had to be 16 years of age or over and residing anywhere in Canada. They had to be either a PLLEX‐C with mental health and/or substance use health research engagement experience or researchers with experience engaging PLLEX‐C in mental health and/or substance use health research. Potential participants were invited to the study from previous Canada‐wide studies on lived experience engagement conducted within the same department, who had provided informed consent to be contacted about future research. In addition, we circulated the study flyer in our networks and shared institutional social media posts (X) with relevant organizations. Individuals who were interested in the study contacted the study staff member by phone, text, or email. The study staff member then screened them for inclusion criteria and sought virtual signed informed consent.

### 2.2 Measures

We used a demographic form to collect basic information about the participants. The form was developed by the researchers based on previous studies and revised with important contributions from the lived experience working group team members. We conducted the focus group and interview discussions using a semi‐structured interview guide, which was built based on the literature [3] and through discussion amongst the scientists and lived experience working group. The data from PLLEX‐C participants and researcher participants were collected using the same semi‐structured interview guide.

### 2.3 Procedure

After providing signed electronic informed consent, participants completed the demographic form on REDCap data management software [22]. We then scheduled the interviews with the researchers immediately based on their schedules. We scheduled the focus group discussions with PLLEX‐C participants sequentially as recruitment and participant schedules allowed. We chose focus groups for PLLEX‐C participants to benefit from the group dynamic when discussing engagement activities. However, since we find that focus groups are unfeasible to schedule with researchers, we chose individual interviews with the researcher sample. The focus groups were held on the WebEx teleconferencing system. Focus groups were between 1 h 17 min and 1 h 26 min of recorded time, while individual interviews ranged from 18 to 53 min. The focus group discussions were facilitated by two Research Analysts (RAs), and individual interviews were facilitated by one of the same two RAs. Discussions were audio recorded using WebEx and a backup handheld recorder. Transcriptions were automatically generated by WebEx, then carefully reviewed and corrected manually by an RA. Focus groups and individual interviews took place between Oct. 30, 2024 and Feb. 12, 2025. Honoraria for participating was a $50 gift card. We received ethical approval from the Research Ethics Board of the Centre for Addiction and Mental Health (CAMH). We conducted this Canada‐wide study out of CAMH, which is a tertiary care teaching and research hospital with a strong lived experience engagement infrastructure in place.

### 2.4 Data Analyses

Codebook thematic analysis was selected as an analytical method [23]. After the completion of all data collection, the research lead, one facilitator, one lived experience team member, and a research analyst familiar with the data set met to develop a codebook focused on areas of untapped potential in participants' engagement experiences. The tentative codes were programmed into NVivo 12 [24] and the RA coded the sum of the transcripts into the codebook. Through regular meetings with the research lead and the lived experience working group, we refined the codebook to reflect the data as they were coded. Illustrative quotes were also selected in collaboration with the lived experience working group. Ongoing working group feedback and attention to reflexivity enhanced the study's rigor and trustworthiness [25].

### 2.5 Lived Experience Engagement

We followed the Patient Engagement in Research conceptual framework [26] and recent best practice guidelines in lived experience engagement [27]. Lived experience engagement in this study is described using recently released guidance for reporting on engagement in research [18]. See Table 1 for the description, which was completed reflexively together with the PLLEX‐C engaged in the study.

**Table 1:** Description of lived/living experience engagement in the study, developed in close collaboration with the PLLEX‐C working group.

Domain	Description
Who was involved?	The PLLEX‐C working group members were full research team members who brought their learned expertise and knowledge base from various disciplines. Their involvement brought lived and living experiences into the research space.
What were the roles, activities and responsibilities?	The engagement process began at study design and continued to manuscript co‐authorship. All members were engaged together and had similar roles. Sample tasks included reviewing ethics board materials (recruitment materials, interview guide, and study flyer), developing the codebook, working together to understand the codes and themes derived from the data, selecting representative quotes, co‐authoring the current manuscript, and supporting related projects such as grant applications and conference submissions, including establishing research questions for a subsequent project.
How did you go about the engagement process?	We scheduled meetings at study onset using a poll. We then developed a terms of reference document to guide the engagement. As per institutional policy, a standard hourly rate was paid for meetings and asynchronous time. We made accommodations as requested, including offering software options, chat and screen options, orienting members to the WebEx technology, working to troubleshoot technical challenges, and offering members various opportunities to provide feedback either verbally or in writing. Despite some challenges and delays, we ultimately shifted from institutionally approved WebEx to Zoom based on working group preference. Accessibility needs were addressed as they arose. Members had choices in how to be engaged.
When did the engagement occur?	The full project, which included a number of sub‐projects, encompassed 19 1‐to‐2 h virtual meetings of the Working Group from overarching project initiation to the initial submission of the current manuscript, plus asynchronous time to review multiple manuscripts emerging from the overarching project. Meetings were held once or twice a month, from April 2024 to the submission of the current manuscript in June 2025.
What was the impact?	Being engaged in the project helped the PLLEX‐C build personal and professional skills. They learned about qualitative research methods, as well as how to balance both the needs of the project and interpersonal interactions in a group setting. They considered it affirming to see their voices and lived/living experiences recognized and valued. They felt they were contributing to improving engagement for PLLEX‐C in research, which was meaningful to them. PLLEX‐C felt that power dynamics were addressed by creating a comfortable environment where they could freely share their insights, they could see their comments being used to edit documents in real time, and the decision‐making process was transparent, with follow‐through. Research staff advanced their understanding of the lived realities of facing PLLEX‐C through reflection and co‐learning. The engagement process grounded the research in real‐world experiences and enhanced its equity focus. The study tools and recruitment materials were made more accessible and relevant to study participants. We conducted an internal evaluation of the engagement process using the Patient Engagement in Research Scale (PEIRS), to continue to improve engagement and to provide the opportunity to offer anonymous feedback on the engagement experience [28].

### 2.6 Research Team Reflexivity

The lead data analyst (JH) is a Chinese female with a Master's degree in Psychology and a full‐time research analyst at CAMH. She is new to lived experience engagement research and willing to learn more through the course of study.

The research lead (LDH) is a white female scientist with a PhD in psychology and an academic position at CAMH. She conducts research on lived experience engagement, and engages PLLEX‐C consistently in her work. She approached this study as both an empirical study and a pragmatic opportunity to learn more about optimal engagement practices. Her positive perspective on engagement may have introduced a bias into the study, although continuing reflexivity and a consistent team‐based approach to the work helped to guard against extensive bias.

The PLLEX‐C Working Group identifies as a mixed group of people with expertise that is defined by lived and living experience, including education, careers, and life trajectories. They have experience in engaging on research projects and are committed to improving the practice of engagement.

## 3 Results

The demographic characteristics of the sample are presented in Table 2. Among the 40 participants, there were 28 PLLEX‐C and 12 researchers/research trainees. The 28 PLLEX‐C participants included 14 who identified as PLLEX and four who identified as family members, and 10 who identified as both PLLEX and family members. While the majority of participants were from Ontario (Canada's largest province), six provinces were represented.

**Table 2:** Demographic characteristics of the sample (*N* = 40).

Characteristic		PLLEX‐C sample (*N* = 28)	Research sample (*N* = 12)
		*n* (%)	*n* (%)
Age	< 30	6 (21.4)	2 (16.7)
	30–59	13 (46.4)	9 (75.0)
	60+	3 (10.7)	0 (0.0)
	Missing	6 (21.4)	1 (8.3)
Gender	Man	8 (28.6)	3 (25.0)
	Woman	18 (64.3)	7 (58.3)
	Transgender/gender nonbinary	1 (3.6)	1 (8.3)
	Missing	1 (3.6)	1 (8.3)
Ethnic/cultural background	White	11 (39.3)	6 (50.0)
	South Asian	7 (25.0)	1 (8.3)
	Black	4 (14.3)	0 (0.0)
	East/Southeast Asian	0 (0.0)	3 (25.0)
	Indigenous Peoples	1 (3.6)	1 (8.3)
	Another background	3 (10.7)	1 (8.3)
	Missing	2 (7.1)	0 (0.0)
Province	Ontario	19 (67.9)	6 (50.0)
	Alberta	3 (10.7)	1 (8.3)
	Manitoba	2 (7.1)	1 (8.3)
	British Columbia	4 (14.3)	1 (8.3)
	Quebec	0 (0.0)	2 (16.7)
	Saskatchewan	0 (0.0)	1 (8.3)
Highest level of education	High school diploma or less	2 (7.2)	0 (0.0)
	Some postsecondary	6 (21.4)	0 (0.0)
	Postsecondary diploma/degree	9 (32.1)	1 (8.3)
	Post‐graduate degree	11 (39.3)	11 (91.7)
Born in Canada	Yes	16 (57.1)	8 (66.7)
	No	11 (39.3)	4 (33.3)
	Missing	1 (3.6)	0 (0.0)
Population of the research in which participants were engaged	Child research	4 (14.3)	1 (8.3)
	Youth research	14 (50.0)	4 (33.3)
	Adult research	18 (64.3)	10 (83.3)
	Geriatrics research	5 (17.9)	0 (0.0)

Participants described multiple areas in which PLLEX‐C were either under‐engaged or engaged in limited ways across different phases of mental health and substance use health research. Our findings are organized into three main areas of engagement: (1) Project Initiation, (2) Project Operations, and (3) End‐of‐Grant Knowledge Translation, each described by subthemes as presented in Table 3. Similar gaps were identified by both PLLEX‐C participants and researcher participants, across the same stages of the research; however, as a whole, PLLEX‐C tended to identify more gaps in engagement, while researchers tended to be more satisfied with the current level of engagement.

**Table 3:** Themes and sub‐themes.

Themes	Subthemes
1. Project Initiation	1a. Establishing research questions and priorities
	1b. Understanding and contributing to the grant application process
	1c. Understanding and contributing to the REB process
2. Research Operations	2a. Recruitment processes
	2b. Data analysis
3. End‐of‐Grant Knowledge Translation	3a. Conference presentations, webinars
	3b. Manuscript writing and co‐authorship

### 3.1 Project Initiation

Participants commonly reported that PLLEX‐C were rarely involved in shaping research questions or priorities during the early stages of research project development. In many cases, PLLEX‐C were invited to join after the research topic, goals, and even content outlines had already been decided upon. Participants felt restricted in their ability to influence research questions or priorities. Their input was often limited to offering suggestions or reacting to existing plans, rather than co‐developing the project vision.

I was not there at the beginning stage of the brainstorming (…). I could only really point out some additional ways that we could use the data that we're trying to compile.

(PLLEX‐C #1, Focus Group)

Despite PLLEX‐C expressing a strong interest in being part of early decision‐making, they described “*nobody was involved*” *[PLLEX‐C #2, Focus Group]* or having limited influence on the conceptualization of projects.

Many participants reported limited or no involvement in writing or shaping grant applications. Even when PLLEX‐C were included, their contributions were often minimal or symbolic, perhaps in part due to research process barriers such as short grant deadlines in contrast with longer timeframes required for authentic engagement.

They're either not involved in the grant submission, or they're involved in the grant submission in a way that's like, ‘yeah, you're on the grant submission, but your contributions meaningfully to shaping that grant submission are fairly negligible’.

(Researcher #1, Interview)

Similar to the missing involvement in establishing research questions and priorities, many PLLEX‐C felt they were brought in after key decisions had already been made, though some expressed interest in contributing to areas such as ‘*How do we put the budget together? What do we put in the application? What do we do for advertising? How do we build preferred flyers?*’ *[PLLEX‐C #3, Focus Group]*. This further reinforced a sense of tokenism, especially for grant applications that formally require the inclusion of patient partners.

Am I being asked to be part of this because they really want my perspective? Or they—just because they need the grant to be approved?

(PLLEX‐C #4, Focus Group)

In some cases, they were invited to provide comments on drafts, but were not part of the core grant writing team, such as shaping the content or structure of the applications.

We are always included after the fact (…) even with the grant writing, they will say ‘please read the grants see if you agree’. And you can have some suggestions, but never did they meet with us before even the grant application with any priority settings.

(PLLEX‐C #5, Focus Group)

Participants described limited to no engagement of PLLEX‐C in the development of research ethics board (REB) applications. REB processes were generally managed solely by research personnel, with little recognition of where or how PLLEX‐C could contribute. Some research sample participants viewed this phase as primarily administrative and did not see it as a space where PLLEX‐C could contribute meaningfully, which contrasted with PLLEX‐C participant perspectives. However, it is unclear whether the researchers are speaking about the administrative aspects of the ethics application like filling out forms, or the many research protocol decisions and materials that go into building a complete ethics submission.

When I think then back on the research ethics, it would not be something that I would prioritize people with lived experience to be involved in (…). It's also not something that I think of global impact on a project and what a large or a meaningful impact would be. This is not an area that I think factors into that. It's just almost what's happening on the administrative side behind the scenes, and I think that you can involve people with lived experience, not in the REB process and protocol development and still have them really shape the project in its outputs.

(Researcher #1, Interview)

Participants were often engaged after REB approval, missing the opportunity to shape ethics‐related decision and ethics‐governed project materials. One participant described this late involvement, noting they ‘*did a lot on this project’*, but hadn't ‘*been involved in a research project where—until they're past ethics’*. *[PLLEX‐C #6, Focus Group]*

In addition, some participants highlighted limited understanding of the REB process itself, including the technical language used and their potential role within it, which points to a shared uncertainty between researchers and PLLEX‐C around how to meaningfully involve lived/living experience in ethics decision‐making.

[The co‐production] It's mostly missing…I think what I see [is] that since people with lived experience are, maybe it's an assumption, they may not have a good understanding, and it's very true that we don't understand the REB process, the terms, the charges that are used in this process. So, there is a lack of more partnership in this process.

(PLLEX‐C #7, Focus Group)

While many participants described limited involvement in early research stages, some researchers shared positive experiences of early engagement, which suggested that early‐stage collaboration is possible in engagement research process.

So… [the working group members] were involved in the drafting of the proposal. They were involved in the drafting of the ethics application.

(Researcher #2, Interview)

### 3.2 Research Operations

While PLLEX‐C were often included as part of the study teams, their involvement in the operational aspects of research was sometimes limited in scope or occurred, again, after key decisions had been made, which may suggest that they were not full study team members. Participants described that this was particularly the case for the recruitment processes and data analysis.

PLLEX‐C had limited opportunities to contribute to recruitment processes, and were often brought in after the decision‐making process was complete. Some participants were involved in the recruitment process by reviewing recruitment posters or refining participant‐facing materials, but they were rarely involved in designing these materials. For example, they described not having the opportunity to decide on the ‘*communication style or method*’ and ‘*the information that it contained*’ from the start. They felt that this area of untapped potential in their contributions highlights the importance of understanding ‘*where it is that you can feel the most impact*’ as a PLLEX‐C *[PLLEX‐C #8, Focus Group]*. Participants generally agreed that PLLEX‐C were underutilized at the participant recruitment stage, although they felt that recruitment was a key area in which they could have valuable input.

But overall my observation is that people with lived experience (…) can contribute a lot in recruitment and participation and dissemination and much beyond that, but they are not utilized effectively because of many gaps in the system.

(PLLEX‐C #7, Focus Group)

Across focus groups and interviews, participants also described that PLLEX‐C were rarely involved in core aspects of data analysis. Participants described that they were involved almost exclusively in qualitative analysis, such as coding and theme development, with little to no participation in quantitative research activities. As one participant mentioned, “*I've only been involved in the data analysis stage for qualitative research. I haven't been in quantitative*.” *[PLLEX‐C #9, Focus Group]* Participants suggested that qualitative methods felt more accessible and aligned with lived or living experience, while quantitative methods were viewed as more technical or “*only be understood by other numerical people in academia*” *[Researcher #3, Interview]*.

Many participants described being brought in after preliminary findings were established by researchers across both qualitative and quantitative research. However, the discussions were largely centered on qualitative work, where they had more experience. Their experience was limited to reviewing qualitative codebooks, validating interpretations, or offering comments on phrasing. Sometimes, their contributions were limited to consultation or were tokenistic in nature.

My impact looked a little bit different, because it was kind of like a confirmation. It was like ‘does this resonate with your experience that you've had?’ So most of my feedback was like ‘Yes’ or ‘No.’ There wasn't a lot of space for me to, like—after all of this stuff has been done, there's not much that I can suggest to do differently.

(PLLEX‐C #2, Focus Group)

Some participants perceived that they were not fully trusted to take part in data analysis. As one participant mentioned, ‘*nobody trusted me to do [qualitative data analysis]*’ *[PLLEX‐C #5, Focus Group]*, and they were usually only invited to comment on the existing analysis framework or offer clarification rather than participate directly in analytic decisions. This may be in part due to institutional barriers such as training requirements, access to secure servers by non‐staff, and unionized roles. Nevertheless, this underutilization appeared to stem also in part from seeing PLLEX‐C as lacking the skills or capacity to handle technical demands.

I have seen in my experience, [people with lived experience] are mostly not involved in data interpretation analysis, and maybe it is based on some assumptions that they may not have a skill set or mean they may not have a capacity to do that, but maybe much beyond.

(PLLEX‐C #7, Focus Group)

Several participants expressed a strong desire for more training in data analysis to enable deeper involvement beyond surface‐level feedback. Training on data analysis is ‘*missing in general with patient engagement research*’ *[PLLEX‐C #4, Focus Group]*, which could be one of the key reasons that PLLEX‐C's contribution to analysis remained limited. By offering opportunities for training, PLLEX‐C can better identify “*nuances*” that researchers might miss, and the research results would be ‘*much richer*’ *[PLLEX‐C #6, Focus Group]*.

I would love to see that [providing training] happening and I would love if they could increase our hours so that we could take on some of that work (…). They [researchers] are not gonna know everything that they are missing when they look at the data, but we with lived experience and family members, we will know those nuances and those little bit of differences.

(PLLEX‐C #6, Focus Group)

Although engagement in research operations was often limited, there were examples of meaningful inclusion and contribution with training and support.

We were taught how to engage. We were taught how to pick out themes, analyze the data, transcribe the data, use grounded theory methodology to process the information.

(PLLEX‐C #1, Focus Group)

### 3.3 End‐of‐Grant Knowledge Translation

Participants reported limited involvement in end‐of‐grant knowledge translation activities, such as conference presentations, webinars, and manuscript writing. They did report some opportunities to review or comment on knowledge dissemination products. However, few PLLEX‐C reported being engaged in deeper roles, such as being listed as co‐authors on manuscripts or reports. PLLEX‐C were generally involved in this end‐of‐grant stage at the level of consultation rather than co‐production, and the process was generally led by researchers.

PLLEX‐C involvement in presentations and dissemination of knowledge products was typically limited to a surface level. While they were invited to take part in knowledge translation presentations, few were engaged in shaping the messaging or making key decisions on the materials.

I think in terms of the changes that were made, they were mostly superficial in terms of like overall design changes to the presentation versus real structural changes in terms of the dissemination of the information that was gathered. And I overall don't think there was a huge change that was made based on the feedback. Like, it wasn't like significant changes, it was more like small design related.

(PLLEX‐C #10, Focus Group)

Participants also mentioned limited contributions to manuscript writing and co‐authorship at this stage. Many participants reported not being included as co‐authors or ‘*not being asked to help’ [PLLEX‐C #11, Focus Group]* with knowledge translation and reporting at all, or not even being certain of the role or process.

I don't recall that [being listed as coauthor] being a thing that came up (…) I think in very much it was left—it was a publication within the health authority rather than academia. And I'm sure my name was on the list of people who are involved in it, but it wasn't such a formal, you know, “this is a published paper and it's out to the university of this and that” thing. It was more of an in‐house program rather than sort of across the board academia in Canada.

(PLLEX‐C #12, Focus Group)

Some participants described that the writing responsibilities of manuscripts often remained with academic team members or students, and PLLEX‐C were ‘*given the chance to look through and make comments*’ *[PLLEX‐C #6, Focus Group]*. However, some researcher participants also mentioned the systemic issues that contributed to such missed opportunities, including the lack of time and resources to support PLLEX‐C's engagement. As a result, manuscript writing or authorship practices were often shaped by traditional academic norms, institutional rules, or assumptions about who is qualified to write.

It comes down to who gets paid to do this study, how much time do I have to train someone? (…) I can't be responsible to train all my graduate students plus everyone else.

(Researcher #2, Interview)

While some participants felt left out of the key dissemination activities, others described contrasting experiences of substantial involvement in this process.

I was involved in the manuscript process and then also a lot of the dissemination through presentations, like poster presentations, but also oral presentations at different conferences and symposiums.

(PLLEX‐C #11, Focus Group)

## 4 Discussion

This qualitative descriptive study aimed to explore the gaps in terms of the contributions that PLLEX‐C make to research projects. We found multiple gaps of engagement throughout the research lifecycle, at the project initiation stage, during research operations, and at end‐of‐grant knowledge translation. These gaps were identified by PLLEX‐C and researchers, but emphasized more strongly by PLLEX‐C. Those participants expressed interest in engaging more fulsomely in the research process, including aspects like priority setting, grant applications, ethics applications, recruitment processes, data analysis, conference presentation, and manuscript writing, as might be expected from full staff roles. Some PLLEX‐C participants had experienced these stages of the research process, showing that more fulsome engagement is feasible. However, for many, these remain areas of untapped potential where more extensive engagement appears to be possible and desirable for many PLLEX‐C contributing to mental health and substance use health research, despite potential barriers experienced by some researchers and research institutions in this area. More active roles across the research lifecycle can help teams move from tokenistic to authentic engagement in which PLLEX‐C make positive impacts on the research conducted, despite barriers that must be addressed.

This study showed that many PLLEX‐C describe areas of untapped potential where they could contribute more, across the lifecycle of the research. The reduced gaps identified by researcher participants showed that these areas of potential contribution may be difficult for researchers to identify, if working according to their usual practices. A first important step is for researchers to discuss PLLEX‐Cs skills and interests with them to identify aspects of the research that they may want to contribute to, recognizing their expertise and investing in skill building. It is important to remember that PLLEX‐C bring not only lived and living expertise to the engagement context, but also educational and professional backgrounds, skills, and interests across disciplines, and may therefore have unexpected areas of potential contribution. Their goals for themselves might be different from the tasks identified by the research staff members. This process should include reflection on the scope of the engagement and expectations for involvement in the project. However, researchers might find that certain areas of engagement are sometimes not possible in the scope of a study due to institutional role definitions, formal academic training requirements, time limitations, and financial and human resources. If this is the case, the lack of engagement in a certain aspect of the study this should be explained and justified in a transparent and upfront manner, in a two‐way conversation to avoid the tokenism that is always a risk [29].

In our study, we found that many PLLEX‐C participants had not had the opportunity to be engaged authentically at the early stages of the project, at the research priority setting, grant application, and ethics board review stages. Previous research has recommended that engagement begin as early as possible in the research lifecycle, but has found that there are barriers to early engagement [10]. The lack of early engagement is a considerable gap, given the critical importance of the early stages of research to defining the work to be done, alongside the real benefits that PLLEX‐C can bring to the relevance of the research [30]. A challenge with this is that priority setting typically occurs before research funding is available, making it difficult to fund engagement at the earliest stages. Small engagement funding allotments might enable researchers to conduct authentic early engagement processes before full project funding, so that PLLEX‐C can meaningfully contribute to establishing the direction of future research.

In line with our findings, some PLLEX‐C might have a keen interest in being involved in either qualitative or quantitative data analysis. This can have many advantages, but can also be challenging for a number of reasons [15]. As non‐staff advisors, PLLEX‐C may not have the institutional authorization to access confidential research data on their secure server; they may not have the academic training required for some analytical approaches, nor the institutional research privacy and ethics training required to handle the data under local ethics requirements. The goal for researchers, then, is not to ask PLLEX‐C to function as institutional biostatisticians, but to recognize that there may be creative ways to include them meaningfully in both qualitative and quantitative data analysis processes that helps them develop their professional skills, while also helping the data analyst understand lived experience perspectives. Qualitative coding can be accomplished together with PLLEX‐C teams. In terms of quantitative data analysis, shadowing a statistician might be a valuable opportunity for skill development by both parties. This line of reflection can be applied to any aspect of the research process in which the PLLEX‐C are interested in being engaged, but the role is not immediately apparent. In doing so together with the PLLEX‐C, it may be possible to find innovative solutions to strengthen the engagement process, capture that untapped potential, and move toward more authentic engagement practices.

End‐of‐grant knowledge translation activities, like manuscript co‐authorship, is another important area of untapped potential worthy of consideration, as reflected in our findings. Recent guidance on PLLEX‐C co‐authorship suggests that PLLEX‐C can be authentically and meaningfully engaged in manuscript writing and that this is often a meaningful experience for many of them [18]. It has been further argued that co‐authorship is a critical part of lived/living experience engaged research [31]. Recommendations and lay authorship guidelines have recently been proposed that may help explain co‐authorship to PLLEX‐C [32], including describing the important role of contributing to a paper as a content contributor and text editor, versus being the individual who actually takes on the bulk of the concrete writing process. Co‐authorship goes hand‐in‐hand with co‐presenting the research at conferences [31], which provides PLLEX‐C the opportunity to develop their public speaking and knowledge dissemination skills and the chance to represent the project in the public eye. Based on the interests of the PLLEX‐C engaged, this should be planned for at the outset, and budgeted for in grant applications to ensure that funds are available to support PLLEX‐C involvement at conferences.

Future research is required to continue evaluating engagement, notably evaluating what PLLEX‐C are actually contributing to research compared to the best practice guidelines for engagement in research [27]. Multi‐method evaluations are required, inclusive of qualitative and quantitative methods, and evaluations should be co‐developed with PLLEX‐C [33]. Strategies to improve engagement across the research lifecycle include pre‐grant funding allotments, leveraging previous grant funding to support priority setting on the next grant, budgeting more in grant applications for engagement, and considering full‐time or part‐time lived experience staff roles. These may help improve engagement, from the earliest stages of protocol development to end‐of‐grant knowledge translation.

### 4.1 Strengths and Limitations

PLLEX‐C were engaged throughout the study, adding to its rigor, trustworthiness, and relevance. We collected the perspectives of both PLLEX‐C and researchers on this issue, to gain a fulsome perspective on the research question. There was diversity in the sample across age, ethic background, and location across Canada. Participants had experience in research across the lifespan. However, the findings cannot be used to make conclusions about specific subgroups of the population, such as Indigenous Peoples, LGBTQ2S+ populations, youth or geriatric populations, or other subgroups of the population. We did not specifically recruit for certain vulnerable populations, such as precariously housed individuals or people with disabilities. In‐depth information on the type of research conducted by the researcher sample participants (qualitative, quantitative, preclinical) is not available, which may have affected the findings. We collected the data using focus groups for PLLEX‐C versus individual interviews for researchers due to pragmatic scheduling reasons, which may have introduced some bias in the data. We combined mental health and substance use health, which is important given the extremely high rates of comorbidity and the historical artificial divide between them in the context of concurrent disorders [34], but which may have missed certain considerations specific to one group. Reflecting the frequent co‐occurrence of mental health and substance use health experiences within families, there were few participants who represented family/caregiver perspectives alone.

## 5 Conclusions

PLLEX‐C are willing to be engaged in research across the research lifecycle, but many have experienced areas of untapped potential. Clear engagement plans should be backed by sufficient project budgets that allow for engagement activities across the entire project, from priority setting to knowledge translation. To develop appropriate creative and flexible plans for authentic engagement in a given project, it is important to have open and honest discussions with the PLLEX‐C engaged, at the start of the study and on an ongoing basis. This will help the researchers understand their areas of skill, interest, and professional development goals, while also helping PLLEX‐C understand any limitations or barriers that might prevent engagement in certain aspects of the project. This can lead to the development of personalized engagement plans that are meaningful to them and maximize their engagement in the project.

## Author Contributions

**Lisa D. Hawke:** conceptualization, methodology, validation, data curation, formal analysis, visualization, writing – original draft, writing – review and editing, supervision, project administration, funding acquisition. **Jingyi Hou:** conceptualization, methodology, validation, formal analysis, resources, writing – original draft, writing – review and editing. **Charlotte Munro:** conceptualization, validation, writing – review and editing. **Claudia Sendanyoye:** conceptualization, validation, writing – review and editing. **Shoshana Hauer:** conceptualization, validation, writing – review and editing. **Mary Rose van Kesteren:** conceptualization, validation, writing – review and editing. **Katie Upham:** conceptualization, validation, writing – review and editing. **Tanya Halsall:** conceptualization, validation, writing – review and editing. **Yona Lunsky:** conceptualization, validation, writing – review and editing.

## Ethics Statement

Research ethics board approval was received from the Centre for Addiction and Mental Health, REB# 2024‐070. All participants provided electronic signed informed consent.

## Conflicts of Interest

The authors declare no conflicts of interest.

## Supporting information

Appendix A.

## Data Availability

The data that support the findings of this study are available on request from the corresponding author. The data are not publicly available due to privacy or ethical restrictions.
